# High-Trough Plasma Concentration of Afatinib Is Associated with Dose Reduction

**DOI:** 10.3390/cancers13143425

**Published:** 2021-07-08

**Authors:** Takayuki Takahashi, Hideyuki Terazono, Takayuki Suetsugu, Hideki Sugawara, Junko Arima, Mina Nitta, Toru Tanabe, Kayu Okutsu, Ryuji Ikeda, Keiko Mizuno, Hiromasa Inoue, Yasuo Takeda

**Affiliations:** 1 Department of Clinical Pharmacy and Pharmacology, Graduate School of Medical and Dental Sciences, Kagoshima University, Kagoshima 890-8520, Japan; t-takah@m3.kufm.kagoshima-u.ac.jp (T.T.); suu@m2.kufm.kagoshima-u.ac.jp (H.S.); junko@m3.kufm.kagoshima-u.ac.jp (J.A.); ushiyama@m3.kufm.kagoshima-u.ac.jp (M.N.); takeda@m.kufm.kagoshima-u.ac.jp (Y.T.); 2 Department of Pulmonary Medicine, Graduate School of Medical and Dental Sciences, Kagoshima University, Kagoshima 890-8520, Japan; taka3741@m2.kufm.kagoshima-u.ac.jp (T.S.); keim@m.kufm.kagoshima-u.ac.jp (K.M.); inoue@m2.kufm.kagoshima-u.ac.jp (H.I.); 3 Sendai Medical Association Hospital, Sendai 895-0005, Japan; drug-info@sendaihp.jp; 4 United Graduate School of Agricultural Sciences, Kagoshima University, Kagoshima 890-0065, Japan; k8673711@kadai.jp; 5 Department of Pharmacy, University of Miyazaki Hospital, Miyazaki 889-1692, Japan; ryuji_ikeda@med.miyazaki-u.ac.jp

**Keywords:** afatinib, epidermal growth factor receptor, tyrosine kinase inhibitor, non-small-cell lung cancer, trough plasma concentration, receiver operating characteristic curve

## Abstract

**Simple Summary:**

Afatinib is used to treat non-small-cell lung cancer (NSCLC) harboring epidermal growth factor receptor (EGFR) mutation as a second-generation EGFR-tyrosine kinase inhibitor (TKI). We examined the relationship between the trough plasma concentrations of afatinib and adverse effects, including diarrhea, rash, stomatitis and mucositis. The dose reduction of afatinib was associated with the trough plasma concentration of afatinib in this paper. As a result, we clarified that a higher trough plasma concentration induced adverse events, and there was a threshold to reduce the dosage or not. Monitoring plasma concentrations of afatinib is useful to predict the adverse effects of afatinib and to support quality of life in patients with EGFR-mutated advanced NSCLC.

**Abstract:**

Afatinib is used to treat non-small-cell lung cancer (NSCLC) harboring epidermal growth factor receptor (EGFR) mutation as a second-generation EGFR-tyrosine kinase inhibitor (TKI). Early prediction of adverse effects based on the pharmacokinetics of afatinib enables support for quality of life (QOL) in patients with no change in efficacy. We examined the pharmacokinetic relationship between trough plasma concentration and adverse effects and evaluated the utility of measuring the trough plasma concentration of afatinib as the first EGFR-TKI treatment for NSCLC in a prospective multicenter study. Twenty-four patients treated with afatinib were enrolled in this study. All blood samples were collected at the trough point, and plasma concentrations were measured using high-performance liquid chromatography–tandem mass spectrometry. Logistic regression analysis for the dose reduction of afatinib was performed, and the receiver operating characteristic (ROC) curve was plotted. Although all patients started afatinib at 40 mg/day, plasma concentrations were variable, and mean and median trough plasma concentrations were 32.9 ng/mL and 32.5 ng/mL in this study, respectively. Minimum and maximum trough plasma concentrations were 10.4 ng/mL and 72.7 ng/mL, respectively. This variability was speculated to involve personal parameters such as laboratory data. However, no patient characteristics or laboratory data examined correlated with the trough plasma concentration of afatinib, except albumin. Albumin showed a weak correlation with plasma concentration (r = 0.60, *p* = 0.009). The trough plasma concentration of afatinib was significantly associated with the dose reduction of afatinib (*p* = 0.047). The area under the ROC curve (AUC) for the trough plasma concentration of afatinib was 0.81. The cut-off value was 21.4 ng/mL. The sensitivity and specificity of the cut-off as a risk factor were 0.80 and 0.75. In summary, the trough plasma concentration of afatinib was associated with continued or reduced dosage because of the onset of several adverse effects, and a threshold was seen. Adverse effects not only lower QOL but also hinder continued treatment. Measuring plasma concentrations of afatinib appears valuable to predict adverse effects and continue effective therapy.

## 1. Introduction

Epidermal growth factor receptor (EGFR) tyrosine kinase inhibitors (TKIs) show robust efficacy for EGFR mutation-positive non-small-cell lung cancer (NSCLC). EGFR-TKIs have been used worldwide as a first-line treatment for patients in this setting [1].

Afatinib is used for the treatment of NSCLC harboring EGFR mutation as a second-generation EGFR-TKI. Unlike the first generation of reversible EGFR-TKIs, including gefitinib and erlotinib, afatinib binds irreversibly to EGFR (ErbB1), ErbB2 (human epidermal growth factor receptor 2 (HER2)), and ErbB4 [2,3]. The prolongation of progression-free survival (PFS) and improvement of symptoms and quality of life (QOL) in EGFR mutation-positive NSCLC treated with afatinib have been shown in several clinical trials [4,5,6,7,8,9,10,11,12,13,14,15]. In a Japanese population, afatinib showed prolonged overall survival (OS) in a subgroup analysis of LUX-Lung 3 [16]. Although afatinib is likely to prove more effective for Japanese patients than for American or European patients according to this subgroup analysis, the frequency and severity of adverse effects, including diarrhea, rash, stomatitis, and mucositis, were worse in Japanese patients than in American or European patients. Over 75% of Japanese patients in the subgroup analysis experienced dose reduction because of the expression of adverse effects.

Some studies have revealed the relationship between the frequency and severity of adverse effects and plasma concentration of afatinib. In a study with a combined analysis of Lux-Lung 3 and 6, higher trough concentration was shown to lead to dose reduction, whereas lower trough concentration led to dose escalation, and no difference in PFS was seen between patients with or without dose reduction [17]. Furthermore, a meta-analysis of population pharmacokinetics revealed some risk factors for a higher area under the curve (AUC) for afatinib [18]. That analysis reported female sex, lower creatinine clearance, higher Eastern Cooperative Oncology Group (ECOG) performance status (PS), alkaline phosphatase (ALP), lactate dehydrogenase (LDH), and total protein were factors associated with a higher AUC. Measuring the blood concentration of afatinib thus allows us to support QOL in patients without changing efficacy.

Measuring the trough plasma concentration is easier and more tolerable than determining the AUC. However, previous studies have only evaluated the effectiveness of afatinib according to the AUC. We therefore investigated the pharmacokinetic relationship between trough plasma concentration and adverse events. Furthermore, we evaluated the utility of measuring the trough plasma concentration of afatinib as the first EGFR-TKI treatment for NSCLC in a prospective multicenter study.

## 2. Materials and Methods

### 2.1. Subjects

The subjects in this study were patients who received afatinib (Giotrif^®^ tablets; Japanese Boehringer Ingelheim Co., Tokyo, Japan) for NSCLC at five centers in Kagoshima, Japan: Kagoshima University Hospital; Kagoshima City Hospital; Minami Kyushu National Hospital; Sendai Medical Association Hospital; and Imakiire General Hospital, from October 2017 to March 2019.

The study protocol was approved by the Ethics Review Boards of Kagoshima University Hospital (Approval Number: 170258) and all other participating centers. All patients provided written informed consent for participation in this study. This study was performed in accordance with the Declaration of Helsinki.

### 2.2. Administration of Afatinib and Blood Sampling

The attending physician started afatinib administration at 40 mg/day. All patients were administered afatinib between meals. Administration was discontinued when adverse effects greater than or equal to grade 3, as defined in Common Terminology Criteria for Adverse Events (CTCAE) version 4.0, were observed. Moreover, when adverse effects were seen to recover to grade 1, the dosage was reduced by 10 mg/day, and afatinib administration was restarted.

All blood samples were collected at the trough point just before the next administration, and blood samples were collected at Days 8–14 from the beginning of afatinib at 40 mg/day.

### 2.3. Chemicals and Reagents

Afatinib was purchased from Toronto Research Chemicals (Toronto, Canada). Imatinib mesylate for use as the internal standard (IS) was purchased from LKT Laboratories (St. Louis, MO, USA). tert-Butyl methyl ether (TBME), ammonium formate, formic acid, and liquid chromatography–mass spectrometry (LC-MS)-grade acetonitrile were purchased from Wako (Osaka, Japan).

### 2.4. Measuring Plasma Concentration of Afatinib in Patients

Collected blood samples were centrifuged at 1300× *g* for 10 min at ambient temperature. We then collected and preserved the supernatant of blood samples from subjects at −80 °C until concentrations of afatinib in plasma were measured.

The sample extraction method reported in 2015 by Hayashi et al. [19] was slightly modified. In summary, internal standard (IS) (5 µL of 2 µM) was added to the collected plasma sample (250 µL) in a polypropylene tube. After mixing, TBME (1.5 mL) was added, and the tube was vortexed for 30 s then centrifuged at 2300× *g* for 10 min at ambient temperature. A sample of the supernatant (1.75 mL) was transferred to a glass tube and dried under nitrogen gas. A mobile phase (50 µL) was added to the dried sample containing glass tubes. After filtration through a 0.2 µm pore membrane filter (GL Chromatodisk; GL Science, Tokyo, Japan), samples were transferred to a 200 µL polypropylene autosampler vial, and a sample (10 µL) was injected onto the LC instrument for quantitative analysis using an autosampler operating at 4 °C.

Plasma concentrations of afatinib were measured in patients using a high-performance LC-MS/MS system (AB SCIEX 3200QTRAP LC-MS/MS; SCIEX, Tokyo, Japan, and LC-20; Shimadzu, Kyoto, Japan). The mobile phase consisted of 2 nm of ammonium formate buffer (pH 4.1) and acetonitrile (65:35, *v/v*). Detection was carried out using multiple reaction monitoring. The lower limit of quantification was 1.67 ng/mL.

### 2.5. Evaluation of Efficacy and Adverse Effects of Afatinib

The attending physician evaluated efficacy in accordance with the Response Evaluation Criteria in Solid Tumors (RECIST) and adverse effects in accordance with the Common Terminology Criteria for Adverse Events (CTCAE) version 4.0.

### 2.6. Correlation between Trough Plasma Concentration and Patient Characteristics, Laboratory Data

Patient characteristics and laboratory data of patients were collected from the database at Kagoshima University Hospital, Minami-Kyushu Hospital, and Sendai Medical Association Hospital. Spearman’s rank correlation test was conducted to confirm the correlation between trough plasma concentration, patient characteristics, and laboratory data.

### 2.7. Logistic Regression Analysis for Dose Reduction of Afatinib

The relationship between trough plasma concentration and dose reduction of afatinib was analyzed by logistic regression analysis. Items showing significant differences were considered risk factors for the dose reduction of afatinib.

### 2.8. ROC Curve

We plotted ROC curves to identify cut-off values of the trough plasma concentration of afatinib for dose reduction. The cut-off value was considered to be the maximum of the sum of sensitivity and specificity.

### 2.9. Statistics

Differences were considered statistically significant at values of *p* < 0.05. Statistical analyses, including *t*-test, Spearman’s rank correlation test, logistic regression analysis, and ROC curve, were performed using JMP^®^ Pro 15 (SAS Institute Inc., Cary, NC, USA).

## 3. Results

### 3.1. Patient Characteristics

The participants in this study comprised 24 NSCLC patients treated with afatinib. The characteristics of patients who experienced a dose reduction of afatinib are summarized in Table 1.

The median age was 67 years (range: 46–79 years), and half of the patients were female (*n* = 12). Twenty of the 24 patients experienced a dose reduction of afatinib in the study. From the physician’s judgment based on RECIST, all patients of the nonreduction group were judged as partial response (PR), while the reduction group had some variety: stable disease (SD), 5 patients; PR, 13; progressive disease (PD), 1 patient; and nonevaluated, 1. Two groups did not show significant differences (Fisher’s exact test *p* = 0.62).

### 3.2. Histogram of Trough Plasma Concentration of Afatinib

**Figure 1:**
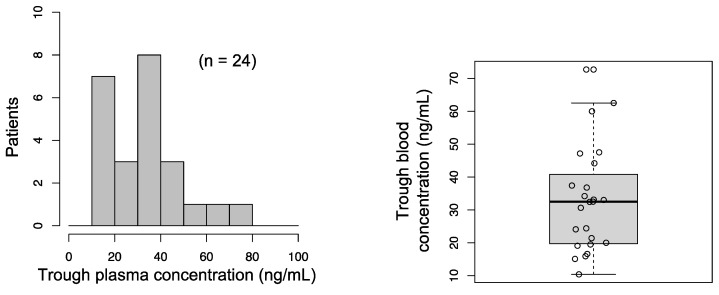
Histogram and boxplot of the trough plasma concentration of afatinib (*n* = 24).

**Figure 2:**
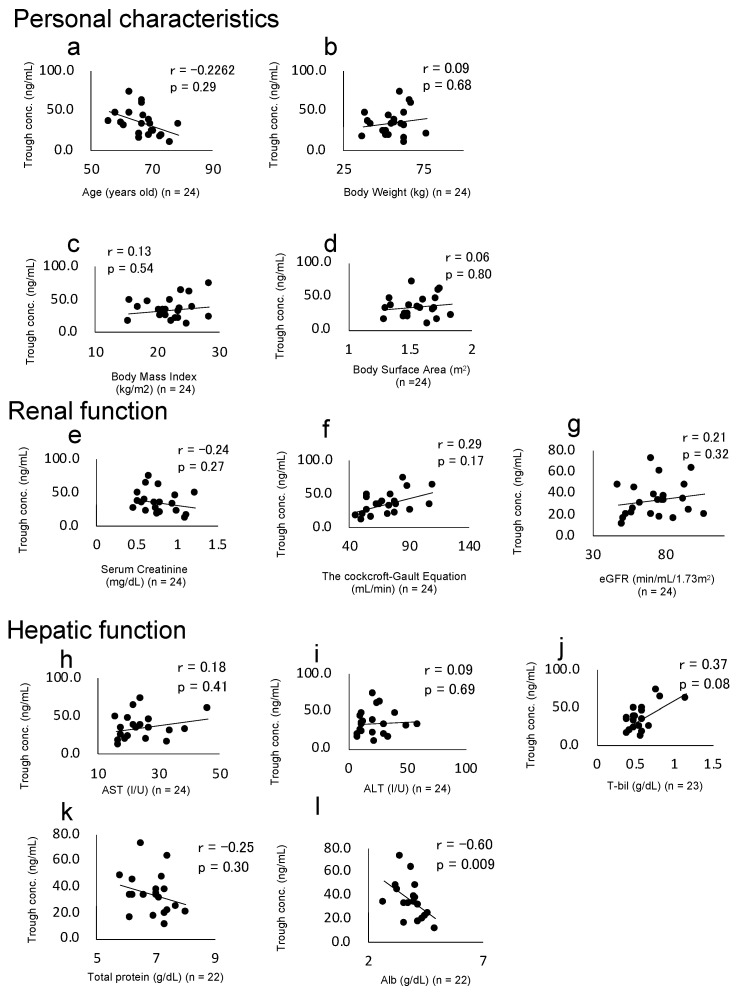
Correlation between the trough plasma concentration of afatinib and laboratory data (personal parameters, renal function, and hepatic function). The r- and *p*-values provided above each graph represent Spearman’s rank correlation and the *p*-value, respectively. (**a**) Age. (**b**) Body weight. (**c**) Body mass index. (**d**) Body surface area. (**e**) Serum creatinine. (**f**) Cockcroft–Gault equation. (**g**) Estimated glomerular filtration rate. (**h**) Aspartate aminotransferase. (**i**) Alanine aminotransferase. (**j**) Total bilirubin. (**k**) Total protein. (**l**) Albumin. (**a**–**d**) Correlations between trough plasma concentration and personal characteristics. (**e**–**g**) Correlations between trough plasma concentration and renal function. (**h**–**l**) Correlations between trough plasma concentration and hepatic function.

A histogram and boxplot of the trough plasma concentration of afatinib are shown in Figure 1, respectively. The mean and median trough plasma concentrations were 32.9 ng/mL and 32.5 ng/mL, respectively. Minimum and maximum trough plasma concentrations were 10.4 ng/mL and 72.7 ng/mL (Figure 2). The standard deviation (SD) was 16.1. The coefficient of variation (CV) was 48.9%, similar to a previous report [17].

### 3.3. Associations between Trough Plasma Concentration, Patient Characteristics, and Laboratory Data

Spearman’s rank correlation test was used to confirm patient characteristics or laboratory data correlating with the plasma concentration of afatinib (Figure 2). Serum albumin was the only factor found to correlate with the trough plasma concentration of afatinib (r = −0.5988, *p* = 0.0087, *n* = 22) (Figure 2l), although serum albumin was not examined in two patients. Other laboratory data, including height, body weight, body surface area, total protein, total bilirubin (T-bil), aspartate aminotransferase (AST), alanine aminotransferase (ALT), serum creatinine, estimated glomerular filtration rate (eGFR), and creatinine clearance, calculated by the Cockcroft–Gault equation, showed no correlation with the trough plasma concentration of afatinib. T-bil was not examined in one patient, and total protein was not examined in two patients.

### 3.4. Trough Plasma Concentration of Afatinib and Logistic Regression Analysis between Continuous Dosage and Dose Reduction

**Figure 3:**
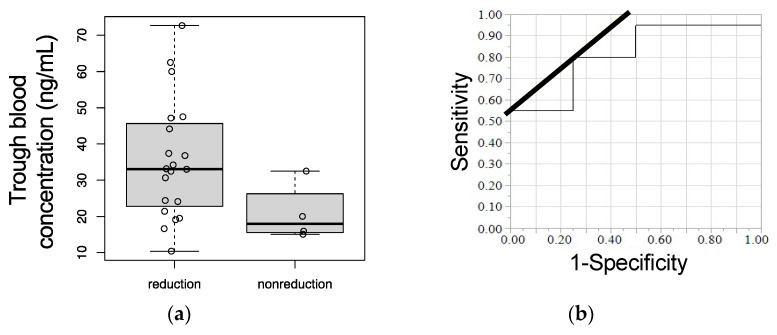
Relationship between the trough plasma concentration of afatinib and continuous dose/dose reduction. (**a**) Boxplot of distribution for the trough plasma concentration of afatinib between continuous dosage and dose reduction. (**b**) Receiver operating characteristic curve for the trough plasma concentration of afatinib.

The median trough plasma concentrations of afatinib identifying dose reduction and continuous dosage were 33.1 and 18.0 ng/mL, respectively (Figure 3a). Logistic regression analysis between dose reduction and trough plasma concentration of afatinib is shown in Table 2. The reasons for the dose reduction of afatinib were diarrhea (*n* = 15, 75% of the reduction group), mucositis oral (*n* = 2, 10% of the reduction group), rash (*n* = 2. 10% of the reduction group), and paronychia (*n* =1, 5% of the reduction group).

The trough plasma concentration of afatinib was significantly associated with the dose reduction of afatinib (*p* = 0.047).

### 3.5. Receiver Operating Characteristic (ROC) Curves

The area under the ROC curve (AUC) for the trough plasma concentration of afatinib was 0.81, and the cut-off value for the trough plasma concentration of afatinib was 21.4 ng/mL (Table 2). The sensitivity and specificity of this cut-off were 0.80 and 0.75, as summarized in Table 2, and the ROC curve is shown in Figure 3b.

## 4. Discussion

A relationship between the dose reduction of afatinib and the high-trough plasma concentration of afatinib was found in Japanese patients receiving afatinib as the first EGFR-TKI. The rate of dose reduction of afatinib and inter-patient variability of the trough plasma concentration of afatinib were high. Measuring the trough plasma concentration of afatinib thus has the potential to predict the dose reduction of afatinib as a first-line treatment for EGFR mutation-positive NSCLC.

In the present study, a relationship between the dose reduction of afatinib and the trough plasma concentration of afatinib was found by logistic regression analysis. The mean trough plasma concentration of patients who experienced a dose reduction was about 1.7 times that of patients who did not experience a dose reduction. In addition, high inter-patient variability of the trough plasma concentration of afatinib was detected, with a CV of 48.94%. This result of variability was in agreement with previous studies in EGFR mutation-positive NSCLC [7,17,20]. Collectively, results from the present study indicate that the therapeutic drug monitoring of afatinib is important to predict dose reduction or the occurrence of adverse effects. In addition, the cut-off value for the trough plasma concentration of afatinib was 21.4 ng/mL in the present study. This value is similar to the mean trough plasma concentration of afatinib after dose adjustment in post hoc analyses of the LUX-Lung 3 and 6 trial [17] and a previous report of Japanese EGFR mutation-positive NSCLC [21]. In light of the results, a cut-off value of 21.4 ng/mL is useful for earlier prediction of the severe adverse effects such as diarrhea, oral mucositis, and rash. If we have this information, we can prepare the medication to avoid or relieve the adverse effects and follow continuous treatments.

The post hoc analyses of the LUX-Lung 3 and 6 trial also discussed efficacy [17]. Both studies examined whether PFS changed among patients who underwent dose reductions within 6 months and those who remained on afatinib ≥ 40 mg/day. The dose reduction of afatinib did not significantly change the estimated PFS (hazard ratios of LUX-Lung 3 and 6 were 1.25 (95% confidence interval (CI): 0.91–1.72; *p* = 0.175) and 1.00 (95%CI: 0.69–1.46; *p* = 0.982)), respectively. Furthermore, trough plasma concentrations of patients who remained on 40 mg/day compared to those who underwent dose reduction were 23.3 ng/mL and 22.8 ng/mL, respectively, in those studies. These results support our own findings and suggest that monitoring trough plasma concentration is effective for avoiding adverse effects and continuing effective therapy.

Serum albumin was the only laboratory data to show a correlation with the trough plasma concentration of afatinib (r = −0.5988, *p* = 0.0087), with data from only two patients not examined. Serum albumin may thus offer an indicator of elevated exposure to afatinib. However, precisely predicting the plasma concentration of afatinib from our result is difficult. Dömötör et al. reported that human serum albumin and afatinib do not appear to show high affinity in the experimental data [22]. The difference between our data and the report is unclear. Further research targeted the relationship between serum afatinib concentration, and serum albumin is essential to predict dose reduction (=severe adverse effect).

Recently, population pharmacokinetics of afatinib in Japanese patients, including those who underwent gefitinib or erlotinib treatments in the past, have shown that hepatic impairment is associated with afatinib pharmacokinetics, and trough plasma concentrations of afatinib on Day 8 were significantly higher in patients who experienced dose reduction or interruption than in those who did not [23]. However, hepatic function tests, including AST, ALT, and T-bil, were not correlated with the trough plasma concentration of afatinib. Moreover, a past study showed that hepatic impairment had no effect on afatinib excretion [24]. Further studies are thus needed to confirm the relationships between hepatic function and afatinib pharmacokinetics.

According to the present study, serum creatine and creatinine clearance as calculated from the Cockcroft–Gault equation did not correlate with the trough plasma concentration of afatinib. However, from a previous report on the population pharmacokinetics of afatinib, the AUC was increased by 27.8% with creatinine clearance of 43 mL/min as calculated from the Cockcroft–Gault equation when compared with creatinine clearance of 79 mL/min [18], although afatinib is mainly (around 85.4%) excreted into feces as afatinib dimalate [25]. In addition, from a case series of Japanese patients, the estimated glomerular filtration rate (eGFR) was significantly lower in patients who experienced a dose reduction of afatinib than patients who did not [21]. Moreover, some reports have shown that other nonrenal elimination pathways were decreased in renal dysfunction [26,27]. The mechanism of the relationship between renal function and adverse effects or the dose reduction of afatinib is not fully understood. Further studies are thus needed to elucidate the reasons for the relationship between renal function and adverse effects or dose reduction of afatinib.

Regarding other TKIs, some studies have indicated a relationship between the plasma concentration and adverse effects of TKIs. The steady-state trough plasma concentration of sunitinib is associated with fatigue and anorexia [28]. In addition, pazopanib, a multikinase inhibitor, shows relationships between steady-state trough plasma concentration and adverse effects, including hypertension, fatigue, and anorexia in renal cell carcinoma [29,30]. These results support the suggestion from the present results that measuring plasma concentrations of afatinib is valuable to predict adverse effects or dose reduction.

Although the present study was a multicenter prospective study, the number of patients was small. This may have contributed to the lack of significant findings in this study. Therefore, it cannot have been ruled out that other factors may have an effect. Further study involving more samples needs to perform multivariate analysis. In the present study, a detailed analysis of concomitant medications was not conducted. Ritonavir has been shown to elevate the plasma concentration and AUC for afatinib [31]. Strong P-glycoprotein inhibitors including ritonavir, itraconazole, and verapamil hydrochloride, or inducers including rifampicin and carbamazepine, are thus likely to affect the plasma concentration of afatinib. As far as prescription records could be confirmed, no patients in this study were treated with strong P-glycoprotein inhibitors or inducers. In this study, the number of the nonreduction group was small because the patients we selected were firstly administered afatinib without previously being administered other TKIs to exclude the other effects. Therefore, this study just reflected the adverse effect of afatinib. The dosage for the patients was reduced from 40 mg to 30 or 20 mg in the LUX-Lung 3 clinical trial. In that study, the ratio of the nonreduction group was 46.7%. It is difficult to collect the patients; however, it is essential to increase the number of patients to resolve the limitation or strengthen the results of the study.

## 5. Conclusions

Dose reduction of afatinib is associated with the trough plasma concentration of afatinib in patients with EGFR-mutated advanced NSCLC receiving afatinib as the first EGFR-TKI treatment. Measuring and monitoring the plasma concentration of afatinib thus appears valuable to predict adverse effects of afatinib and to support QOL in patients with EGFR-mutated advanced NSCLC.

## Figures and Tables

**Table 1:** Characteristics of patients in the study.

All Patients (*n* = 24)	
Female, *n* (%)	12 (50)
Age, years, median (range)	67 (46−79)
Height, cm, median (range)	158.9 (145.2−177.8)
Body weight, kg, median (range)	57.4 (37.2−77.1)
Body mass index, kg/m^2^, median (range)	22.7 (15.27−28.49)
Body surface area, m^2^, median (range)	1.58 (1.30−1.84)
EGFR mutation, *n* (%)	
Exon 19del	9 (37.5)
L858R	15 (62.5)
ECOG performance status, *n* (%)	
0	12 (50)
1	12 (50)
Stage, *n* (%)	
Postoperative recurrence	3 (12.5)
III B	3 (12.5)
IV	18 (75)
Dose reduction of afatinib, *n* (%)	
Reduction	20 (83.3)
Nonreduction	4 (16.7)

ECOG, Eastern Cooperative Oncology Group; EGFR, epidermal growth factor receptor.

**Table 2:** Logistic regression analysis for the dose reduction of afatinib and the parameters of the receiver operating characteristic (ROC) curve.

*p*-Value	Area under the ROC Curve	Cut-off for Trough Plasma Concentration (ng/mL)	Sensitivity	Specificity
0.0472	0.8125	21.4	0.80	0.75

## Data Availability

The data presented in this study are available on request from the corresponding author.

## References

[1] Gelatti A.C., Drilon A., Santini F.C. (2019). Optimizing the sequencing of tyrosine kinase inhibitors (TKIs) in epidermal growth factor receptor (EGFR) mutation-positive non-small cell lung cancer (NSCLC). Lung Cancer.

[2] Li D., Ambrogio L., Shimamura T., Kubo S.H., Takahashi M., Chirieac L.R., Padera R.F., Shapiro G.I., Baum A., Himmelsbach F. (2008). BIBW2992, an irreversible EGFR/HER2 inhibitor highly effective in preclinical lung cancer models. Oncogene.

[3] Solca F., Dahl G., Zoephel A., Bader G., Sanderson M., Klein C., Krämer O., Himmelsbach F., Haaksma E., Adolf G.R. (2012). Target Binding Properties and Cellular Activity of Afatinib (BIBW 2992), an Irreversible ErbB Family Blocker. J. Pharmacol. Exp. Ther..

[4] Miller V.A., Hirsh V., Cadranel J., Chen Y.-M., Park K., Kim S.-W., Zhou C., Su W.-C., Wang M., Sun Y. (2012). Afatinib versus placebo for patients with advanced, metastatic non-small-cell lung cancer after failure of erlotinib, gefitinib, or both, and one or two lines of chemotherapy (LUX-Lung 1): A phase 2b/3 randomised trial. Lancet Oncol..

[5] Hirsh V., Cadranel J., Cong X.J., Fairclough D., Finnern H.W., Lorence R.M., Miller V.A., Palmer M., Yang J.C.-H. (2013). Symptom and Quality of Life Benefit of Afatinib in Advanced Non–Small-Cell Lung Cancer Patients Previously Treated with Erlotinib or Gefitinib: Results of a Randomized Phase IIb/III Trial (LUX-Lung 1). J. Thorac. Oncol..

[6] Yang J.C.-H., Shih J.-Y., Su W.-C., Hsia T.-C., Tsai C.-M., Ou S.-H.I., Yu C.-J., Chang G.-C., Ho C.-L., Sequist L.V. (2012). Afatinib for patients with lung adenocarcinoma and epidermal growth factor receptor mutations (LUX-Lung 2): A phase 2 trial. Lancet Oncol..

[7] Sequist L.V., Yang J.C.-H., Yamamoto N., Obyrne K., Hirsh V., Mok T., Geater S.L., Orlov S., Tsai C.-M., Boyer M. (2013). Phase III Study of Afatinib or Cisplatin Plus Pemetrexed in Patients With Metastatic Lung Adenocarcinoma With EGFR Mutations. J. Clin. Oncol..

[8] Yang J.C.-H., Hirsh V., Schuler M., Yamamoto N., O’Byrne K.J., Mok T., Zazulina V., Shahidi M., Lungershausen J., Massey D. (2013). Symptom Control and Quality of Life in LUX-Lung 3: A Phase III Study of Afatinib or Cisplatin/Pemetrexed in Patients With Advanced Lung Adenocarcinoma With EGFR Mutations. J. Clin. Oncol..

[9] Katakami N., Atagi S., Goto K., Hida T., Horai T., Inoue A., Ichinose Y., Koboyashi K., Takeda K., Kiura K. (2013). LUX-Lung 4: A Phase II Trial of Afatinib in Patients With Advanced Non–Small-Cell Lung Cancer Who Progressed During Prior Treatment With Erlotinib, Gefitinib, or Both. J. Clin. Oncol..

[10] Schuler M., Yang J.C.-H., Park K., Kim J.-H., Bennouna J., Chen Y.-M., Chouaid C., De Marinis F., Feng J.-F., Grossi F. (2016). Afatinib beyond progression in patients with non-small-cell lung cancer following chemotherapy, erlotinib/gefitinib and afatinib: Phase III randomized LUX-Lung 5 trial. Ann. Oncol..

[11] Wu Y.-L., Zhou C., Hu C.-P., Feng J., Lu S., Huang Y., Li W., Hou M., Shi J.H., Lee K.Y. (2014). Afatinib versus cisplatin plus gemcitabine for first-line treatment of Asian patients with advanced non-small-cell lung cancer harbouring EGFR mutations (LUX-Lung 6): An open-label, randomised phase 3 trial. Lancet Oncol..

[12] Geater S.L., Xu C.-R., Zhou C., Hu C.-P., Feng J., Lu S., Huang Y., Juliane L., Hou M., Shi J.H. (2015). Symptom and Quality of Life Improvement in LUX-Lung 6: An Open-Label Phase III Study of Afatinib Versus Cisplatin/Gemcitabine in Asian Patients With EGFR Mutation-Positive Advanced Non–small-cell Lung Cancer. J. Thorac. Oncol..

[13] Yang J.C.-H., Wu Y., Schuler M., Sebastian M., Popat S., Yamamoto N., Zhou C., Hu C.-P., O’Byrne K., Feng J. (2015). Afatinib versus cisplatin-based chemotherapy for EGFR mutation-positive lung adenocarcinoma (LUX-Lung 3 and LUX-Lung 6): Analysis of overall survival data from two randomised, phase 3 trials. Lancet Oncol..

[14] Park K., Tan E.-H., O’Byrne K., Zhang L., Boyer M., Mok T., Hirsh V., Yang J.C.-H., Lee K.H., Lu S. (2016). Afatinib versus gefitinib as first-line treatment of patients with EGFR mutation-positive non-small-cell lung cancer (LUX-Lung 7): A phase 2B, open-label, randomised controlled trial. Lancet Oncol..

[15] Schuler M., Wu Y.-L., Hirsh V., O’Byrne K., Yamamoto N., Mok T., Popat S., Sequist L.V., Massey D., Zazulina V. (2016). First-Line Afatinib versus Chemotherapy in Patients with Non–Small Cell Lung Cancer and Common Epidermal Growth Factor Receptor Gene Mutations and Brain Metastases. J. Thorac. Oncol..

[16] Kato T., Yoshioka H., Okamoto I., Yokoyama A., Hida T., Seto T., Kiura K., Massey D., Seki Y., Yamamoto N. (2015). Afatinib versus cisplatin plus pemetrexed in Japanese patients with advanced non-small cell lung cancer harboring activatingEGFRmutations: Subgroup analysis of LUX-Lung 3. Cancer Sci..

[17] Yang J.C.-H., Sequist L.V., Zhou C., Schuler M., Geater S.L., Mok T., Hu C.-P., Yamamoto N., Feng J., O’Byrne K. (2016). Effect of dose adjustment on the safety and efficacy of afatinib for EGFR mutation-positive lung adenocarcinoma: Post hoc analyses of the randomized LUX-Lung 3 and 6 trials. Ann. Oncol..

[18] Freiwald M., Schmid U., Fleury A., Wind S., Stopfer P., Staab A. (2014). Population pharmacokinetics of afatinib, an irreversible ErbB family blocker, in patients with various solid tumors. Cancer Chemother. Pharmacol..

[19] Hayashi H., Kita Y., Iihara H., Yanase K., Ohno Y., Hirose C., Yamada M., Todoroki K., Kitaichi K., Minatoguchi S. (2016). Simultaneous and rapid determination of gefitinib, erlotinib and afatinib plasma levels using liquid chromatography/tandem mass spectrometry in patients with non-small-cell lung cancer. Biomed. Chromatogr..

[20] Murakami H., Tamura T., Takahashi T., Nokihara H., Naito T., Nakamura Y., Nishio K., Seki Y., Sarashina A., Shahidi M. (2011). Phase I study of continuous afatinib (BIBW 2992) in patients with advanced non-small cell lung cancer after prior chemotherapy/erlotinib/gefitinib (LUX-Lung 4). Cancer Chemother. Pharmacol..

[21] Sato J., Morikawa N., Chiba R., Nihei S., Moriguchi S., Saito H., Yamauchi K., Kudo K. (2017). Case series on the association between blood levels and side effects of afatinib maleate. Cancer Chemother. Pharmacol..

[22] Dömötör O., Pelivan K., Borics A., Keppler B.K., Kowol C.R., Enyedy É.A. (2018). Comparative studies on the human serum albumin binding of the clinically approved EGFR inhibitors gefitinib, erlotinib, afatinib, osimertinib and the investigational inhibitor KP2187. J. Pharm. Biomed. Anal..

[23] Nakao K., Kobuchi S., Marutani S., Iwazaki A., Tamiya A., Isa S., Okishio K., Kanazu M., Tamiya M., Hirashima T. (2019). Population pharmacokinetics of afatinib and exposure-safety relationships in Japanese patients with EGFR mutation-positive non-small cell lung cancer. Sci. Rep..

[24] Schnell D., Buschke S., Fuchs H., Gansser D., Goeldner R.-G., Uttenreuther-Fischer M., Stopfer P., Wind S., Petersen-Sylla M., Halabi A. (2014). Pharmacokinetics of afatinib in subjects with mild or moderate hepatic impairment. Cancer Chemother. Pharmacol..

[25] Stopfer P., Marzin K., Narjes H., Gansser D., Shahidi M., Uttereuther-Fischer M., Ebner T. (2012). Afatinib pharmacokinetics and metabolism after oral administration to healthy male volunteers. Cancer Chemother. Pharmacol..

[26] Nolin T.D., Naud J., Leblond F.A., Pichette V. (2008). Emerging Evidence of the Impact of Kidney Disease on Drug Metabolism and Transport. Clin. Pharmacol. Ther..

[27] Zhang Y., Zhang L., Abraham S., Apparaju S., Wu T.-C., Strong J.M., Xiao S., Atkinson A.J., Thummel K.E., Leeder J.S. (2008). Assessment of the Impact of Renal Impairment on Systemic Exposure of New Molecular Entities: Evaluation of Recent New Drug Applications. Clin. Pharmacol. Ther..

[28] Noda S., Otsuji T., Baba M., Yoshida T., Kageyama S., Okamoto K., Okada Y., Kawauchi A., Onishi H., Hira D. (2015). Assessment of Sunitinib-Induced Toxicities and Clinical Outcomes Based on Therapeutic Drug Monitoring of Sunitinib for Patients with Renal Cell Carcinoma. Clin. Genitourin. Cancer.

[29] Suttle A.B., Ball H.A., Molimard M., Hutson T., Carpenter C.M., Rajagopalan D., Lin Y., Swann S.L., Amado R.G., Pandite L. (2014). Relationships between pazopanib exposure and clinical safety and efficacy in patients with advanced renal cell carcinoma. Br. J. Cancer.

[30] Noda S., Yoshida T., Hira D., Murai R., Tomita K., Tsuru T., Kageyama S., Kawauchi A., Ikeda Y., Morita S.-Y. (2019). Exploratory Investigation of Target Pazopanib Concentration Range for Patients With Renal Cell Carcinoma. Clin. Genitourin. Cancer.

[31] Wind S., Giessmann T., Jungnik A., Brand T., Marzin K., Bertulis J., Hocke J., Gansser D., Stopfer P. (2014). Pharmacokinetic Drug Interactions of Afatinib with Rifampicin and Ritonavir. Clin. Drug Investig..

